# Predictive and prognostic properties of TB-LAM among HIV-positive patients initiating ART in Johannesburg, South Africa

**DOI:** 10.11604/pamj.2015.22.4.6075

**Published:** 2015-09-03

**Authors:** Alexander d'Elia, Denise Evans, Lynne McNamara, Rebecca Berhanu, Ian Sanne, Elisabet Lönnermark

**Affiliations:** 1 Department of Infectious Diseases, Sahlgrenska University Hospital, Gothenburg, 40032, Sweden; 2 Health Economics and Epidemiology Research Office, Department of Internal Medicine, School of Clinical Medicine, Faculty of Health Sciences, University of the Witwatersrand, Johannesburg, 2193, South Africa; 3 Clinical HIV Research Unit, School of Clinical Medicine, Faculty of Health Sciences, University of the Witwatersrand, Johannesburg, 2193, South Africa; 4 Right To Care, Helen Joseph Hospital, Perth Road, Johannesburg, 2092, South Africa

**Keywords:** Antiretroviral therapy, HIV, lipoarabinomannan, Mycobacterium, TB LAM, South Africa

## Abstract

While the diagnostic properties of the TB LAM urine assay (LAM) have been well-described, little is known about its predictive and prognostic properties at ART initiation in a routine clinic setting. We describe the predictive and prognostic properties of LAM in HIV-positive patients initiating ART at an urban hospital in Johannesburg, South Africa. Retrospective study of HIV-positive adults (>18 years) who initiated standard first-line ART between February 2012 and April 2013 and had a LAM test at initiation. In HIV-positive patients with no known TB at ART initiation, we assessed the sensitivity, specificity and positive/negative likelihood ratios of LAM to predict incident TB within 6 months of ART initiation. In addition, in patients with a TB diagnosis and on TB treatment <3 months at ART initiation, we measured the CD4 response at 6 months on ART. Of the 274 patients without TB at ART initiation, 65% were female with median CD4 count of 213 cells/mm^3^. Among the 14 (5.1%) patients who developed active TB, none were urine LAM +ve at baseline. LAM had poor sensitivity (0.0% 95% CI 0.00-23.2) to predict incident TB within 6 months of initiation. We analyzed 22 patients with a confirmed TB diagnosis at initiation separately. Of these, LAM +ve patients (27%) showed lower CD4 gains compared to LAM negative patients (median increase 103 vs 199 cells/mm^3^; p = 0.08). LAM has limited value for accurately predicting incident TB in patients with higher CD4 counts after ART initiation. LAM may help identify TB/HIV co-infected patients at ART initiation who respond more slowly to treatment and require targeted interventions to improve treatment outcomes. Larger studies with longer patient follow-up are needed.

## Introduction

Tuberculosis (TB) is estimated by the World Health Organization (WHO) to affect about one third of the world population globally [1]. Up to 70% of people with active TB disease are HIV positive and TB is the primary cause of HIV-related mortality in sub-Saharan Africa [1–3]. Several studies have shown that TB co-infection increases the risk of HIV progression and death, particularly if the HIV disease is untreated [3]. Efficient TB screening at baseline is associated with a considerable reduction in the risk of incident TB and associated deaths during the initial months of treatment with ART [4]. If ART is started with active TB infection untreated, patients are at risk of developing immune reconstitution inflammatory syndrome (IRIS). This is a condition resulting from rapid restoration of immune responses to opportunistic infections, causing either deterioration of a treated infection or presentation of a previously sub-clinical infection and can result in significant morbidity [5]. TB in patients initiating ART is frequently undiagnosed [6]. We therefore proposed to determine the predictive and prognostic value of the LAM assay in a routine clinic setting, to assess the risk of incident TB in patients initiating ART who may go untreated for active TB infection.

The recently introduced Alere Determine TB-LAM assay (Determine^™^ TB-LAM urine lateral flow test, Alere Inc., Waltham, MA, USA) is an ELISA-based urine sample test that detects lipoarabinomannan (LAM), a glycolipid found in the cell wall of Mycobacterium tuberculosis. Compared with current diagnostic methods, detection of LAM antigens in urine has several advantages including that the samples are simple to collect, process and store. The test is inexpensive, has a short turnaround time and can be performed at point of care without expensive equipment and highly trained personnel [7]. Recent work has highlighted the importance of adequate point-of-care methods of diagnosis so that patients can be treated at local clinics when first presenting for treatment [8]. Since these clinics often lack resources, simplicity and affordability are very important [7, 9]. The LAM assay works best in HIV-infected patients with advanced immunosuppression where it can help diagnose TB and reduce treatment delay [5, 9] but performs very poorly as a diagnostic test in patients with a CD4 count >200 cells/mm^3^. While the diagnostic properties of LAM have been well-described, little is known about other potential benefits such as the predictive and prognostic properties of routine use at ART initiation in resource limited settings. Recent studies have shown that LAM may be a valuable tool to monitor anti-TB therapy response and have investigated whether LAM results before and after treatment are predictive of mortality [10]. If LAM is reliable it may be useful in identifying those at risk of IRIS, specifically in those initiating ART but who do not receive treatment for active TB. We aimed to determine if LAM at ART initiation could accurately predict the development of incident TB within the first 6 months of ART in HIV positive patients. In addition, we measured the CD4 response at 6 months on ART in patients with a confirmed TB diagnosis at ART initiation and on anti-TB treatment for <3 months (<90 days), compared by LAM result at ART initiation.

## Methods

This was a retrospective analysis of data from a prospective cohort of HIV-infected adults initiating ART at the Themba Lethu Clinic (TLC), Johannesburg, South Africa. The clinic Cohort has been described elsewhere [11]. Patients at TLC are initiated on ART and are routinely followed-up according to the South African National Department of Health (SA DoH) ART guidelines [12, 13]. At ART initiation, the patient is screened for TB and, if positive, referred to the TB Focal Point (TBFP), adjacent to the HIV clinic, for further investigation (chest radiology, Xpert MTB/RIF, sputum microscopy and/or culture). If TB is diagnosed, TB and ART treatment are initiated and followed up at the TBFP. To decrease the risk of IRIS, ART is generally started 2-4 weeks after TB treatment initiation.

Urine samples were collected from 356 HIV + ve adults at ART initiation (t_0_) for LAM testing. Patient demographic and clinical characteristics were similar to those of the larger TLC cohort [11]. Subjects were HIV-positive ART naïve adults (≥18 years of age) initiated according to SA DoH ART treatment guidelines onto a standard first-line regimen of tenofovir (TDF), stavudine (d4T) or zidovudine (AZT) with lamivudine (3TC) and either efavirenz (EFV) or nevirapine (NVP) between February 2012 and April 2013. Duplicate LAM results were excluded, as were patients who did not meet the eligibility criteria. Only the t_0_ result was used and any follow-ups were removed. This left 344 participants in the analysis.

**Figure 1:**
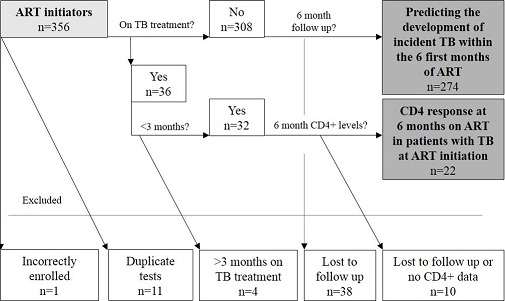
Schematic representation showing the patients included. A total of 356 patients had a TB LAM test at ART initiation. Twelve patients were excluded and the remaining 344 included in the analysis. Two hundred and seventy four patients were not on TB treatment at ART initiation (t_0_) and had a minimum follow-up time of 6 months after ART initiation while 22 patients had been on TB treatment for less than 90 days prior to ART initiation and had a CD4 count recorded at 6 months after ART initiation

For each sub-analysis the following criteria were used: predicting development of incident TB within the 6 first months of ART: (i) No TB treatment at ART initiation (t_0_) and (ii) a minimum follow-up time of 6 months on ART (n = 274) (Figure 1). The aim was to determine the predictive value of the LAM assay in a routine clinic setting in HIV + ve patients with active TB at ART initiation but who remained untreated, so as to determine the risk of incident TB in these patients. We therefore excluded patients with active TB at ART initiation. CD4 response at 6 months on ART in patients with TB at ART initiation: (i) TB treatment for less than 90 days prior to ART initiation and (ii) CD4 count recorded at 6 months after t_0_ (n = 22).

### Study definitions

A positive LAM test was defined as a urine test result of equal or greater intensity than the grade 2+ cut-point [14, 15]. If there was less or no reaction, the result was considered negative. TB at ART initiation (t_0_) was defined as having been on therapy for a period of <90 days prior to ART initiation. Incident TB was defined as a new TB diagnosis (condition and start date) or record of TB treatment (drugs and start date) in any of the study databases within 6 months after t_0_ (<180 days). Incident TB was diagnosed by smear microscopy, culture, Xpert MTB/RIF, radiology and clinical features.

### Data collection

Longitudinal clinical and laboratory data was obtained from the National Health Laboratory Service (NHLS), the TB Focal Point Information System (FIS) and the electronic patient management system, TherapyEdge-HIV^™^, at the site. LAM tests were performed in the Department of Internal Medicine at the University of the Witwatersrand and results, dates and CD4 count at t_0_ and 6 months were recorded. In addition, we reviewed paper record files from Helen Joseph Hospital.

### Statistical analysis

Descriptive analysis and summary statistics were performed with SPSS v22 statistical software. Patient demographics and characteristics were summarized using medians with interquartile range and proportions for categorical variables. LAM result was dichotomized as positive or negative. CD4 at t_0_ was divided into categories of ≤50, 50 - 100, 100 - 200, 200 - 350 and >350 cells/mm^3^. Log binomial regression analysis was used to test the association between gender and CD4 category at t_0_ and LAM status. Among patients with recently diagnosed TB prior to ART initiation, the median time difference (days) between diagnosis and t_0_ was calculated and compared by LAM result. Median and interquartile range was calculated for CD4 at TB treatment initiation (for use in sub-analysis), t_0_ and at 6 months and both CD4 and CD4 increase (continuous variables) were compared by LAM using the Mann-Whitney U test. A p value <0.05 was considered statistically significant.

Predicting development of incident TB within the 6 first months of ART: to assess the predictive properties of LAM for incident TB, we determined the sensitivity, specificity and likelihood ratios of LAM (positive or negative) at ART initiation to correctly predict patients who developed incident TB within 6 months of ART. Additionally, we conducted a sensitivity analysis in patients with no confirmed incident TB. Using the Mann-Whitney U test, we compared the median 6 month CD4 increase by LAM results (false positives vs. true negatives) to see if there were any differences, possibly suggesting undisclosed TB cases.

CD4 response at 6 months on ART in patients with TB at initiation: the Mann-Whitney U test was used to test if there was a difference in the CD4 response from t_0_ to 6 months post-ART initiation, as measured by the median CD4 increase, stratified by LAM at t_0_. The same was used to compare the CD4 count at TB treatment initiation and the median time between TB treatment initiation and t_0_ across the LAM dichotomous variable to ensure that the groups did not differ significantly before t_0_.

Approval for the study was obtained from the Human Research Ethics Committee (HREC) of the University of the Witwatersrand (Clearance certificate M10418). Written informed consent was obtained from all participants.

## Results

The majority of the 274 patients without TB at ART initiation were female, and this was similar between the positive (67%) and negative LAM (65%) groups (p = 0.932). The median CD4 count and age at ART initiation were 213 cells/mm^3^ (IQR 115 - 285) and 37.8 years (IQR 32.3 - 44.3) respectively (Table 1). Only 44% (4/9) of those with a positive LAM and 28% (74/265) with a negative LAM reported any TB symptoms at ART initiation, including cough, fever, night sweats, weight loss and fatigue (p = 0.281). In 54.7% (150/274) of patients the CD4 count was >200 cells/mm^3^. Compared to those with a negative LAM, more patients with a positive LAM had a CD4 count ≤50 cells/mm^3^ (22.2% vs. 11.3%). Conversely, more patients with a negative LAM had a CD4 count >200 cells/mm^3^ (55.5% vs. 33.3%). Consequently, patients with a CD4 count ≤50 cells/mm^3^ were more likely to have a positive LAM result compared to those with a CD4 count >200 cells/mm^3^ (Relative Risk (RR) 2.4; 95% CI 0.77 - 7.25; p = 0.134), although the estimate lacked precision and the confidence interval was wide.

**Table 1:** Characteristics of patients at TB-LAM testing (n = 274)

		All	TB-LAM positive N = 9	TB-LAM negative N = 265
Gender, Female, n (%)		179 (65.3%)	6 (66.7%)	173 (65.3%)
Age, years, median (IQR)		37.8 (32.3 - 44.3)	39.4 (33.0 - 46.3)	37.6 (32.0 - 44.2)
CD4 cells/mm^3^	Median (IQR)	213 (115 - 285)	117 (43 - 260)	214 (120 - 285)
	<50	32 (11.7%)	2 (22.2%)	30 (11.3%)
	51 - 100	30 (10.9%)	1 (11.1%)	29 (10.9%)
	101 - 200	62 (22.6%)	3 (33.3%)	59 (23.3%)
	201 - 350	126 (46.0%)	2 (22.2%)	124 (46.8%)
	≥350	24 (8.8%)	1 (11.1%)	23 (8.7%)
Outcome	Confirmed active TB, n (%)	14 (5.1%)	0 (0%)	14 (5.3%)
	Pulmonary, n (% of TB)	8 (57.1%)	0 (0%)	8 (57.1%)
	Extra pulmonary, n (% of TB)	6 (42.9%)	0 (0%)	6 (42.9%)

### Predicting the development of incident TB within the 6 first months of ART

Of the 274 patients, 9 (3.3%) had a positive LAM result at ART initiation and none were diagnosed with TB within 6 months (sensitivity 0.0%, 95% CI 0.0 - 23.2; specificity 96.5%, 95% CI 93.5 - 98.4; negative likelihood ratio 1.04, 95% CI 1.01 - 1.06) (Table 1). Of the 265 patients who were LAM negative, 14 (5.3%) developed incident TB. Our study did not have sufficient power to detect a significance difference between LAM positive and LAM negative at ART initiation and the development of incident TB (RR 0.92 95% CI 0.06 - 14.3; p = 0.951). The median time from ART initiation to TB diagnosis was 22.5 days (IQR 12.8 - 69.8). Of the 14 file-diagnosed TB cases, 8 (57.1%) were pulmonary while 6 (42.9%) were extra pulmonary. In patients with incident TB, the median CD4 count at ART initiation was 117 cells/mm^3^ (IQR 43.0 - 260.0), and 324 cells/mm^3^ (IQR 177.5 - 376.3) after 6 months; the difference between ART initiation and 6 months was 116 cells/mm^3^ (IQR 74.3 - 198.5). Three of the 14 patients (21.4%) with TB had a CD4 count ≤50 cells/mm^3^; 2 (14.3%) had a CD4 count 51-100 cells/mm^3^; 4 (28.6%) 101-200 cells/mm^3^ and 5 (35.7%) >200 cells/mm^3^. In patients with no diagnosis of incident TB (n = 260), the median CD4 count at ART initiation was 207 cells/mm^3^ (IQR 108.8 - 270.3) and 306 cells/mm^3^ (IQR 202.0 - 425.3) at 6 months. The median increase between ART initiation and 6 months was 130 cells/mm^3^ (IQR 57.5 - 202.5).

### CD4 response at 6 months on ART in patients with TB at ART initiation

Twenty two patients were included in the analysis, of whom 63.6% were female with a median age of 35.1 years (IQR 31.7 - 38.4) (Table 2). At TB treatment initiation, median CD4 count for LAM positive (n = 6) was 68 cells/mm^3^ (IQR 22.3 - 238.5) compared to 144 cells/mm^3^ (IQR 66.3 - 175.3) (p = 0.49) for LAM negative (n = 16). At t_0_ the median difference in CD4 between the 2 groups was 70 cells/mm^3^ (positive 72 cells/mm^3^ IQR 28.5 - 223.5 vs. negative 142 cells/mm^3^ IQR 62.0 - 175.3; p = 0.88). The median time from TB treatment initiation to t_0_ was 28.0 days (IQR 19.3 - 49.3) for the LAM positive group and 21.5 days (IQR 14.5 - 51.5) for the negative group (p = 0.51). The main outcome was the CD4 increase during the first 6 months of ART, compared by LAM result. At 6 months on ART, patients who were positive at ART initiation increased their median CD4 count to 152 cells/mm^3^ (IQR 133.0 - 274.3) compared to 316 cells/mm^3^ (IQR 178.5 - 425.3) in the negative group. At all points LAM positives had lower absolute CD4 counts than negatives (TB treatment initiation -23 cells/mm^3^; t_0_ -11.3 cells/mm^3^ and 6 months -96 cells/mm^3^). Patients who were LAM positive had a lower CD4 gain (103 cells/mm^3^ IQR 18.5 - 149.0) compared to the LAM negatives (199 cells/mm^3^ IQR 89.3 - 271.3), a difference of 96 cells/mm^3^ (p = 0.08). The difference in 6 month CD4 increase between males and females was 82 cells/mm^3^ (p = 0.37), lower among males.

**Table 2:** Characteristics of TB-LAM patients with recently diagnosed active TB (n = 22)

		All	TB-LAM positive N = 6	TB-LAM negative N = 16
Gender, Female, n (%)		14 (63.6%)	4 (66.7%)	14 (63.6%)
Age, years, median (IQR)		35.1 (31.7 - 38.4)	32.0 (31.7 - 36.2)	35.4 (31.9 - 39.1)
CD4 cells/mm^3^, median (IQR)	TB treatment initiation	142 (45.0 - 199.3)	68 (22.3 - 238.5)	144 (66.3 - 175.3)
	ART initiation	141 (30.8 - 177.8)	72 (28.5 - 223.5)	142 (62.0 - 175.3)
	6 months on ART	257 (143.8 - 407.8)	152 (133.0 - 274.3)	316 (178.5 - 425.3)
Time between TB treatment initiation and ART initiation, days, median (IQR)		21.5 (16.8 - 42.5)	28.0 (19.3 - 49.3)	21.5 (14.5 - 51.5)
Outcome - CD4 increase (cells/mm^3^), median (IQR)	ART initiation to 6 months on ART	149 (79.5 - 225.5)	103 (18.5 - 149.0)	199 (89.3 - 271.3)

## Discussion

The diagnostic properties of LAM have been well-described but little is known about its predictive and prognostic properties at ART initiation. We aimed to see if screening with LAM at ART initiation among HIV patients could predict incident TB diagnosis within 6 months. We analyzed CD4 response in the first 6 months of treatment in a sub-group of 22 patients who had already begun TB treatment at ART initiation to see if a positive test resulted in a poorer immunological response to ART. We hypothesized that incident TB diagnosed within 6 months of ART initiation might be present in an active and detectable form at initiation. A positive LAM test could then enable targeted interventions such as more aggressive investigations for TB and, in the case of immunosuppressed patients with undiagnosed TB, predict IRIS. However, results showed that LAM had poor sensitivity for this. The low cost and ease of use of LAM means general screening could be justified if the disease-dismissing properties were adequate. The high specificity is somewhat misleading as prevalence of disease was low. The negative likelihood ratio was virtually 1, indicating that a negative test added no information. A possible explanation for the poor predictive properties could be that incident TB observed during the 6 months was not detectable at t_0_. However, the results were generally concordant with earlier diagnostic studies such as that by Lawn and colleagues, where LAM had a sensitivity of 4% in patients with CD4 >200 cells/mm^3^ when compared to a positive TB sputum culture [9]. This supports the poor performance of LAM in our study where >50% of the patients had a CD4 >200 cells/mm^3^. Nine patients were LAM positive but did not develop incident TB. It is possible that these were false positives due to cross-reactions with other proteins in the urine; 1 patient had a positive symptom screen 1 month prior to ART initiation, however, there was no indication that the patient started TB treatment and 3 patients had tests registered in NHLS but were all negative.

To determine if a positive LAM predicted a poorer immunological response to ART among patients who had been on TB treatment for 3 months or less at ART initiation, we compared LAM test results and CD4 levels at initiation of TB treatment and ART. The numbers were small but we nevertheless demonstrate a noticeable difference in CD4 gain at 6 months post ART initiation, with higher gains observed in those with a negative test (p = 0.08). At all points, LAM positives had lower absolute CD4 counts than LAM negatives. This could be interpreted as meaning that a more aggressive TB and/or poorer response to TB/ART treatment correspond to a poorer CD4 response. Those with incident TB also showed lower gains in CD4 at 6 months post-ART compared to those without (-28 cells/mm^3^). Studies have reported that prevalent or incident TB does not compromise immunological or virological response to ART [16], however these studies did not stratify by LAM at ART initiation. A study from western Kenya showed active TB to be associated with poorer clinical outcomes in HIV-infected African patients on ART. In this study, although TB patients showed an almost identical rise in CD4 count after ART initiation to those with no TB, the overall CD4 count at 1 year was lower among patients with active TB (251 vs 269 cells/µl) [17]. Other possible reasons for the observed trend may be TB therapy-ART interactions, co-morbidity to TB in other diseases and poor adherence to ART or TB therapy.

**Limitations:** Patients already on TB treatment at ART initiation were excluded and, instead, formed the sample for the second objective. The prevalence of disease in the predictive study was, therefore, low (5.1% 95% CI 2.82 - 8.43%). This differs from previous reports from the same clinic (n = 21,101; 11.9% prevalent TB) [11] and a study on 6 month IRIS by Hermans and colleagues in a similar setting that measured a prevalence of 8.8% [18]. Other reasons for the low prevalence could be that patients at TLC are initiated on ART at a higher CD4 count than average (median 213 cells/mm^3^ IQR 115 - 285), and are, thus, generally healthier. Also, since TLC is a part of Helen Joseph Hospital, the sickest patients are initiated on ART in the wards instead of at TLC. The primary limitation of this study is missing data on TB testing at baseline. Among those that were urine LAM positive, there may have been an underlying active TB infection. The screening for TB may have missed some patients with active disease. From the main cohort (n = 356), 13 (3.7%) patients had died, 35 (9.8%) were lost from care and 23 (6.5%) had transferred out so it was not possible to ascertain the impact on mortality or if any cases of active TB were missed. Data regarding Isoniazid Preventive Therapy (IPT) was not available. During the study period, 14% of patients were lost to follow up, limiting the follow-up time for diagnosis of incident TB. Finally, it is possible that not all TB cases were identified during data collection.

## Conclusion

We concluded that LAM has limited value for predicting incident TB accurately in patients with higher CD4 counts after ART initiation. In this study we demonstrated that LAM may help identify TB/HIV co-infected patients at ART initiation who respond more slowly to treatment and require targeted investigations (e.g. drug interactions, adherence counselling, adverse events, treatment failure etc.). Larger studies with longer patient follow-up are required to further assess relations and co-factors, understand why patients who are LAM positive at ART initiation have lower CD4 gains and determine if the inexpensive LAM test can be added to the HIV/TB armoury to improve treatment outcomes.
